# A Physiological Sunday

**Published:** 1889-07-20

**Authors:** T. G. Headley


### A Physiological Sunday.

By the Rev. T. G. Headley.

It is a common experience for young voluntary teachers in Sunday schools when they advance in life to discontinue their teaching, because they have found that whilst they had been assuming the position of teachers they had need of being taught themselves.

And it may be the case with ourselves, also, that whilst we are attempting to teach others we at the same time are learning that we have need of greater accuracy in explaining the meaning of the terms we use. And we are led to this conclusion because we find that some of our readers are rather perplexed to understand what is meant by the term "physiological" when we seem (to them) to speak of it as being something apart from and outside of religion, and that religion was something apart from science; for it is urged that religion is *ars artium, scientia scientarum* (the art of all arts, and the science of all sciences), and therefore it is urged that it would be a contradiction to speak of religion as being foreign to, or opposed to, science and physiology, when religion is itself a science and as much a part of man and Nature as instinct in dogs, etc., is part of their nature, which can be largely developed by training.

And we readily admit the need of more explicitly defining the meaning of the term "physiological," and that when the meaning of the term is fully understood it includes religion, and therefore ought not to be used as though it was something distinct and separate from religion, because the literal and true meaning of "physiology" is the study of Nature. And as the human being called "man" is as much a part of the complex body called "Nature" as any other of the constituent parts which collectively represent Nature, therefore the physiological study of man comprises within its range the religion of man equally as much as the physiological study of a dog, etc., must include, to be a complete study, the instinct of the dog.

For a man implies a human being with a body and a mind, and a sound mind in a sound body; as a man without a mind would be a corpse, so that the mind is as much a necessary part of the being called man as the body; and then it is seen that the study of man's nature, which is called "physiology," must include the study of the mind as well as of the body. And as religion is as much a part of man's nature as instinct in dogs is part of their nature, therefore the physiological study of man must, to be complete, include the study of man's religion.

And the very fact that the nations of the world are divided and opposed to one another in their ideas of what is good and true religion, and that even the people of our own nation who profess to accept one and the same religion are divided and opposed to one another, proves the need there is of investigating and defining what is good and true religion.

For whilst religion divides and separates men from one another instead of uniting them, it would seem possible that what commonly passes current as religion is a spurious article; because the very fact that men are divided and opposed to one another upon what they severally call religion, indicates that error (which is akin to disease) must have crept in to poison the mind like as diseases creep in which poison and kill the body. And as the complete man requires that he should be sound in mind as well as in body, therefore a physiological study of man must as much include the study of the mind as of the body; because the body cannot be well whilst the mind is ill at ease and diseased, as it inevitably must be whilst nations are not only opposed to nations, and Churches to Churches, but even the family under one roof is often divided within and against itself to the destruction of all peace and happiness, though abounding in all else that is needed to make the world an earthly paradise.

And, therefore, instead of religion being something which is foreign to and outside of a physiological study, it is, on the contrary, as much necessary to study it in order to find out what is good and true religion for the health of the mind, as it is necessary to study what is good for the health of the body whilst the body is ill at ease or diseased; because the health of the one is so intermixed with the health of the other it is impossible to separate them.

And whilst even our own Churches are witnessed to be at war with one another, it would be impossible to assert that there is no religious error or disease needing careful diagnosis and skilled removal.

